# Supplementary material for: Development and validation of a risk prediction model for severe postoperative complications in elderly patients with hip fracture
Source: PLoS One. 2024 Nov 13;19(11):e0310416. doi: 10.1371/journal.pone.0310416 (PMC11560009; doi:10.1371/journal.pone.0310416)
Supplement: Attachment — Submitted filename: Response to additional Editor comments.doc [file pone.0310416.s003.doc]

Dear Editor,

I appreciate your valuable input. I will make the necessary revisions as per your guidance and eagerly await your next communication.

This is a retrospective cohort with its inherent weaknesses but provides original data on the utility of risk prediction in a vulnerable group of patients. The authors have revised their paper to strengthen the discussion.

Answer:Thank you for your comments, I will strengthen the discussion.
